# Nuclear Antigens and Auto/Alloantibody Responses: Friend or Foe in Transplant Immunology

**DOI:** 10.1155/2013/267156

**Published:** 2013-04-14

**Authors:** Toshiaki Nakano, Chao-Long Chen, Shigeru Goto

**Affiliations:** ^1^Graduate Institute of Clinical Medical Sciences, Chang Gung University College of Medicine, 123 Ta-Pei Road, Niao-Sung, Kaohsiung 833, Taiwan; ^2^Liver Transplantation Program and Division of Transplant Immunology, Center for Translational Research in Biomedical Sciences, Kaohsiung Chang Gung Memorial Hospital, 123 Ta-Pei Road, Niao-Sung, Kaohsiung 833, Taiwan; ^3^Iwao Hospital, 3059-1 Kawakami, Yufu, Oita 879-5102, Japan

## Abstract

In addition to cellular immune responses, humoral immune responses, mediated by natural antibodies, autoantibodies, and alloantibodies, have increasingly been recognized as causes of organ transplant rejection. In our previous studies, we have demonstrated the induction of antinuclear antibodies against histone H1 and high-mobility group box 1 (HMGB1), in both experimental and clinical liver transplant tolerance. The active induction of antinuclear antibodies is usually an undesirable phenomenon, but it is often observed after liver transplantation. However, the release of nuclear antigens and its suppression by neutralizing antibodies are proposed to be important in the initiation and regulation of immune responses. In this review article, we summarize the current understanding of nuclear antigens and corresponding antinuclear regulatory antibodies (Abregs) on infection, injury, inflammation, transplant rejection, and tolerance induction and discuss the significance of nuclear antigens as diagnostic and therapeutic targets.

## 1. Introduction

Transplantation of cells, tissues, or organs is now widely used to cure patients with life-threatening diseases or traumatic injuries. Except for the use of self-derived grafts or grafts from an identical twin, allograft rejection can be observed acutely and/or chronically [1, 2]. In the current practice of transplantation, the administration of immunosuppressants, such as tacrolimus (FK506) and cyclosporin A, is indispensable for the prevention of allograft rejection [3]. However, the use of these immunosuppressants has limitations, including the necessity of long-term medication and serious side effects, such as nephrotoxicity [4], cardiovascular toxicity [5], and cancer [6]. Therefore, the development of safer and more effective immunosuppressants as well as useful diagnostic tools for the prediction of rejection is an important subject for further improvement of the quality of life of patients and their families after transplantation.

Since the early days of experimental and clinical liver transplantation, it has been known that this organ does not always obey the normal rules of transplant rejection (Medawar's rule of transplantation); for example, all grafts are rejected between unrelated individuals, and the survival rate following liver transplantation is higher than that following the transplantation of other organs [7, 8]. In Dark Agouti (DA) donor livers transplanted into Piebald Virol Glaxo (PVG) recipients, allograft rejection is spontaneously overcome after orthotopic liver transplantation (OLT), resulting in a state of long-lasting and donor-specific tolerance without pharmacological immunosuppression, although PVG recipients acutely reject skin, heart, and renal grafts from DA rats [9]. Interestingly, PVG recipients bearing DA livers could accept skin, heart, and kidney transplants from the DA donor rats but rejected them from third-party strains of rats [10, 11]. The molecular and cellular basis of liver transplant tolerogenicity has not been fully elucidated, but the unique repertoires of nonparenchymal cells including liver antigen-presenting cells (e.g., dendritic cells (DCs), Kupffer cells, and liver sinusoidal endothelial cells) and unconventional lymphoid cells (e.g., NK cells, B-1 cells, and *γδ* T cells), which are rarely present in the blood, may explain the immune privilege of the liver [12]. Our recent study also suggested that mast cells in the donor grafts may play important roles in the induction/maintenance of immune tolerance and liver regeneration, resulting in the replacement of hepatic cells from donor to recipient [13]. In addition, several humoral factors in the serum of a rat tolerogenic OLT model have been identified as immunosuppressive factors, including donor-soluble MHC class I molecules [14], antidonor MHC class II antibodies [15], liver suppressor factor-1 (LSF-1; 40 kDa) [16, 17], LSF-2 (87 kDa), and LSF-3 (10 kDa) [18]. However, most of these humoral factors are found only in the experimental OLT model, and it is hard to translate the findings of this animal study to clinical practice.

In the past decade, we further evaluated humoral factors, specifically IgG antibodies, which are immediately elevated and maintained at a higher level even after the recipients accept the donor liver allografts and demonstrated strong immunosuppressive activity *in vitro* [19, 20]. The screening of autoantigens recognized by immunosuppressive IgG antibodies in the post-OLT sera revealed the spontaneous induction of antinuclear antibodies against histone H1 and high-mobility group box 1 (HMGB1), both in the DA-PVG natural tolerance model and in a patient with operational tolerance [19–22].

In this review article, we summarize the current understanding of nuclear antigens and corresponding antinuclear regulatory antibodies (Abregs) on infection, injury, inflammation, transplant rejection, and tolerance induction and discuss the significance of nuclear antigens as diagnostic and therapeutic targets.

## 2. Induction of Humoral Immune Responses after Transplantation: Link to Rejection or Tolerance?

In the past, organ transplant rejection and tolerance were believed to be mediated almost exclusively by cellular immune responses. Although improvements in T-cell-directed immunosuppression have decreased the incidence of acute cellular rejection, humoral immune responses, mediated by natural antibodies, autoantibodies, and alloantibodies, have increasingly been recognized as causes of organ transplant rejection [23, 24]. The overall incidence of antibody-mediated rejection (AMR) is estimated to be 20%–30% for renal transplant recipients [25]. However, AMR is mainly discussed in ABO blood type-incompatible liver transplantation [26]. Natural antibodies against A/B carbohydrate determinants are likely to develop as a result of exposure to environmental bacteria that express similar determinants. The response of the B-cell compartment to environmental antigens/microbial products and autoantigens has been thought to be derived preferentially from the activation of CD5^+^ B-1 cells. Therefore, CD5^+^ B-1 cells have been speculated to be the major B-cell subset responding to A/B determinants in both mice and humans [27–29]. B-1 cells are present in low numbers in the lymph nodes and spleen and are instead found predominantly in the peritoneal and pleural cavity [30, 31]. Recent reports suggest that splenic CD1d^hi^CD5^+^ B cells are potent regulatory cells that produce IL-10 in models of contact hypersensitivity and experimental allergic encephalomyelitis [32, 33]. Furthermore, Moritoki et al. reported that B cells or B-cell subsets may affect the induction and function of regulatory T cells (Tregs) as suppressors of the T-cell component [34]. Chhabra et al. recently reported the prevention of autoimmune diabetes and the prolongation of islet allograft survival by the administration of naturally occurring IgM autoantibodies [35]. These findings strongly suggest that the induction of natural antibodies or autoantibodies may play an important role in immune regulation and tolerance induction after transplantation.

In our previous studies, we have demonstrated the induction of antinuclear antibodies against histone H1 and HMGB1 both in a rat tolerogenic OLT model and in a patient with operational tolerance [19–22]. In the field of liver transplantation, the induction of autoantibodies (e.g., antinuclear antibody, smooth muscle antibody, and liver-kidney microsomal antibody) has often been observed, particularly in pediatric recipients [36], while the incidence of de novo autoimmune hepatitis in children with elevated serum autoantibodies and liver function tests, hypergammaglobulinemia, and liver pathology showing necroinflammatory disease and fibrosis has been found to be just 1%–7% [37–39]. We also confirmed the significance of antinuclear antibody for protection and recovery from the concanavalin A-(Con A-) induced liver injury mimic of autoimmune hepatitis [40]. Therefore, the induction of autoantibodies in most recipients after liver transplantation may not be associated with any clinical manifestations of autoimmune disorders. A recent study also demonstrated that the long-term administration of tacrolimus to liver transplant recipients induces the production of antinuclear antibodies, whereas the autoimmune disease susceptibility of recipients treated with tacrolimus has not been elucidated [41]. The active induction of antinuclear antibodies is usually an undesirable phenomenon, but why is it often observed after liver transplantation? Is it linked to the immune privilege of the liver? The answers to these questions are still uncertain, but we speculate that the existence of antinuclear antibodies against histone H1 and HMGB1, which possess strong immunosuppressive activity in the systemic circulation, may regulate uncontrollable immune responses such as acute/chronic rejection after transplantation. In other words, the induction of antinuclear antibodies may be a “lethal weapon” to escape the breakdown of our immune system at least in transplant immunology. Our hypothesis is supported by Barnay-Verdier et al., who recently demonstrated that autoantibodies against HMGB1 are produced during sepsis and are associated with a favorable outcome in patients with septic shock [42].

## 3. Nuclear Antigens and Immunogenicity

Why are antinuclear antibodies against histone H1 and HMGB1 elevated in the specific condition of liver transplantation, and do they act as Abregs? An initial mechanism for the induction of antinuclear antibodies is the release of nuclear antigens, and the primary source of nuclear antigens would be damaged hepatic cells due to peritransplant ischemia/reperfusion injury and posttransplant rejection. Specifically, hepatic cell death by necrosis, apoptosis, and autophagy during cold ischemia and warm reperfusion during the course of liver transplantation triggers liver graft dysfunction [43–45]. Indeed, the release of nuclear antigens and its suppression by neutralizing antibodies are proposed to be important in the initiation and regulation of immune responses. HMGB1 is a ubiquitous and abundant chromatin component, and it is currently well known as one of the damage-associated molecular pattern molecules (DAMPs) interacting with the receptor for advanced glycation end product (RAGE), toll-like receptor (TLR)2, TLR4, and TLR9 [46]. Wang et al. first reported the proinflammatory role of HMGB1 in endotoxin lethality in mice and in septic patients [47]. Since then, the proinflammatory roles of HMGB1 in the pathogenesis of many diseases have been reported, including acute lung inflammation [48], atherosclerosis and restenosis after vascular damage [49], severe acute pancreatitis [50], rheumatoid arthritis [51], pulmonary fibrosis [52], stroke [53], Kawasaki disease [54], cold ischemia/reperfusion-induced inflammation [55], liver fibrosis [56], systemic inflammatory response syndrome [57, 58], febrile seizures [59], hyperlipidemia [60], preeclampsia [61], and acute-on-chronic liver failure [62].

However, the roles of histones in immune responses are poorly understood in comparison with HMGB1. Histone H1 has been reported to possess various important functions including a role in transmitting apoptotic signals from the nucleus to the mitochondria, which release apoptogenic factors into the cytoplasm, following DNA double-strand breaks [63] and in normal DC differentiation, based on evidence demonstrating that the production and differentiation of DCs in histone H1^0^-deficient mice are significantly reduced [64]. Our previous study has demonstrated that the translocation of histone H1 from the nucleus to the cytoplasm and the release of their own histone H1 are necessary for DC maturation and the T-cell activation [65]. This function is also similar to the role of HMGB1 in DC maturation [66]. In addition, recent work has clearly demonstrated the induction of inflammatory responses by extracellular histones from dying cells via TLR2 and TLR4 in acute kidney injury [67].

**Figure 1:**
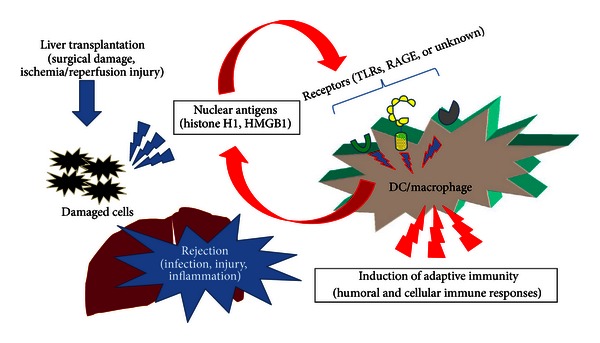
Histone H1 and HMGB1 as “nuclear weapons” in innate and adaptive immune responses. Nuclear antigens released from damaged cells or actively secreted from activated immune cells such as DCs and macrophages are important initiators for the activation of humoral and cellular immune responses during peritransplant ischemia/reperfusion injury and posttransplant rejection as well as infection, injury, and inflammation.

Taken together, these results strongly suggest the significance of nuclear antigens such as histones and HMGB1 that are released from damaged cells or actively secreted from activated immune cells such as DCs and macrophages in the initiation of immune responses during rejection as well as infection, injury, and inflammation (Figure 1). We speculate that the sensitivity to nuclear antigens (i.e., easy production of antinuclear Abregs) may be one of the key factors determining the acceptance or rejection of donor liver allografts [68]. To be exact, antinuclear antibodies include both auto- and alloantibodies due to the different sources of antigens (liver allografts: alloantigens, immune cells: autoantigens) in the case of liver transplantation. In this review article, however, we have defined the induction of antinuclear antibodies as an autoimmune response due to the homological similarity of nuclear antigens even in different species.

## 4. Nuclear Antigens as a Prognostic Marker for Rejection

**Figure 2:**
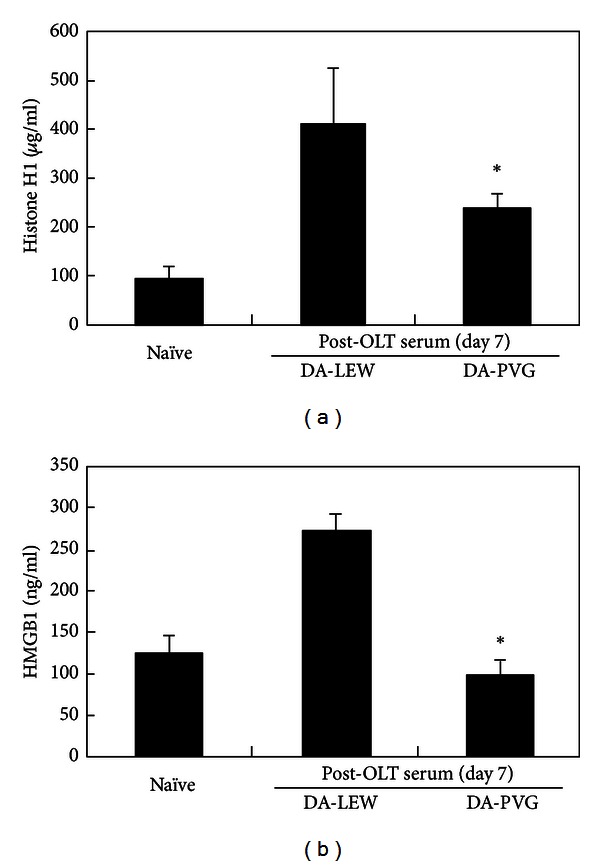
The elevation of circulating histone H1 and HMGB1 during the rejection phase after OLT. The levels of histone H1 and HMGB1 in naive or post-OLT sera were determined by ELISA. For the quantitative determination of histone H1, 0.1 *μ*g of antihistone H1 polyclonal antibody (Santa Cruz Biotechnology, Santa Cruz, CA, USA) in 100 mM NaHCO_3_ (pH 9.3) was coated onto a 96-well microtiter plate (Nalge Nunc International, Roskilde, Denmark) by overnight incubation at 4°C. The plate was then blocked with SuperBlock T20 (PBS) Blocking Buffer (Thermo Fisher Scientific Inc., Rockford, IL, USA), and serum samples (each *n* = 3) (50 *μ*L, ×25 dilution with 10 mM Tris-HCl (pH 8.0), 0.9% (w/v) NaCl, 0.5% (w/v) Tween 20) were added to the wells. Calf thymus histone H1 (Upstate, Charlottesville, VA, USA) was used as a standard. The mixture was incubated at room temperature for 1 hr. Antihistone H1 monoclonal antibody (×500 dilution; Abcam, Cambridge, MA, USA) was then added, and the mixture was incubated at room temperature for 1 hr. Peroxidase-conjugated anti-mouse IgG (×2,000 dilution; Santa Cruz Biotechnology) was then added, and the mixture was incubated at room temperature for 1 hr, followed by the addition of 1-Step Ultra TMB substrate solution (Thermo Fisher Scientific Inc.). For the quantitative determination of HMGB1, a rat HMGB1 ELISA kit (MyBioSource Inc., San Diego, CA, USA) was used according to the manufacturer's protocol. The absorbance (450 nm) was then measured using a Victor X4 Multilabel Plate Reader (PerkinElmer, Shelton, CT, USA). The results are expressed as the mean of three individuals ± SD. *Significantly different compared with the DA-LEW combination (*P* < 0.01, Student's *t*-test).

**Figure 3:**
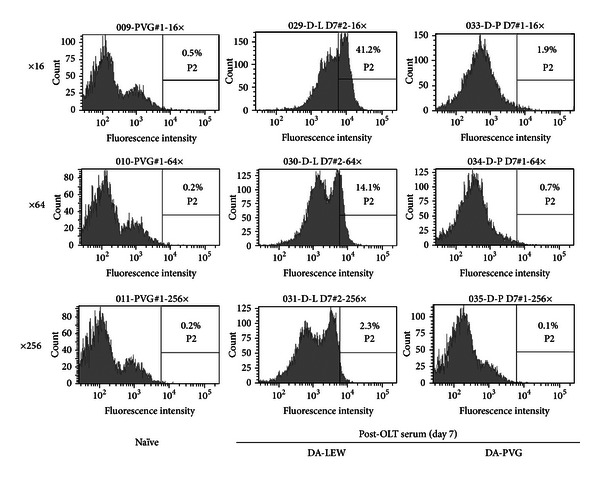
Alloantibody response during the rejection phase after OLT. The alloantibody response was measured by flow cytometry on a single cell suspension of DA rat splenocytes. Briefly, 50 *μ*L aliquots containing 5 × 10^5^ splenocytes was incubated with 50 *μ*L of diluted naive or post-OLT sera (1 : 16, 1 : 64, 1 : 256) for 45 min at 4°C. The washed cells were reacted with 50 *μ*L of a mixture of FITC-conjugated goat antibody specific for the Fc portion of rat IgG (×100 dilution) (Jackson ImmunoResearch Laboratories, West Grove, PA, USA) in PBS containing 1% BSA and 0.02% NaN_3_. After staining, the cells were washed, fixed, and analyzed using an LSRII flow cytometer (BD Biosciences, San Jose, CA, USA). Histograms (representative of three individuals) show the percentage of DA splenocytes recognized by alloantibody (IgG) in the post-OLT sera at day 7 after OLT.

**Figure 4:**
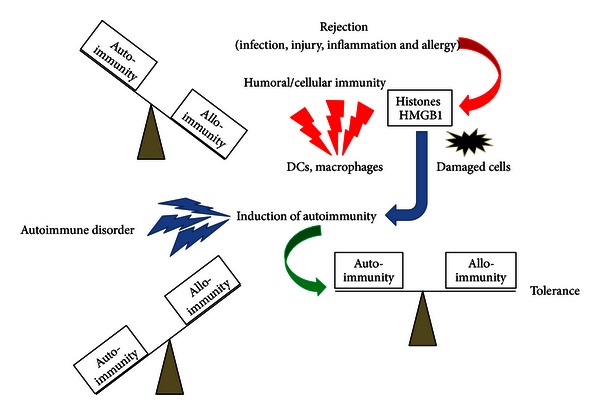
The balance of autoimmunity and alloimmunity (against alloantigens, pathogens, or allergens) is important for immune regulation. During the rejection phase (or when suffering from infection, injury, or allergy), alloimmunity is predominant, and nuclear antigens such as histones and HMGB1 are released from damaged cells, tissues, or organs or are actively secreted from activating immune cells such as DCs and macrophages. The induction of autoimmunity against nuclear antigens (i.e., induction of antinuclear Abregs) may regulate the balance and induce immunological tolerance. Excessive activation of autoimmunity may cause autoimmune disorders.

The release of nuclear antigens into the blood stream has been associated with the progression of several diseases. Hatada et al. reported the elevation of plasma HMGB1 levels in patients with infectious diseases, malignancies, and traumas and suggested that HMGB1 is a potentially suitable prognostic marker of organ failure or disseminated intravascular coagulation [69]. The serum level of HMGB1 in patients with nonsmall cell lung cancer (NSCLC) was significantly higher compared to patients with chronic obstructive pulmonary disease, suggesting that HMGB1 may be a useful marker for evaluating NSCLC progression [70]. A positive association between the circulating HMGB1 level and cardiovascular mortality or traumatic brain injury has also been reported [71, 72]. As shown in Figure 2, the elevation of circulating histone H1 and HMGB1 was confirmed during the rejection phase (day 7) after OLT in a rat acute rejection combination (DA-LEW). However, mild or no elevation of circulating histone H1 and HMGB1 was confirmed in a rat tolerogenic combination (DA-PVG), suggesting the diagnostic potential for the prediction of acute rejection after transplantation. In our previous studies, we have confirmed the induction of humoral immune responses against histone H1 and HMGB1 only in the DA-PVG combination [21, 22], suggesting the blockade of the exposure of nuclear antigens by the induction of corresponding antinuclear Abregs. The induction of antinuclear Abregs could also suppress alloantibody production during the rejection phase (day 7) after OLT (Figure 3). Therefore, the balance between autoimmunity and alloimmunity is important for the prolongation of allograft survival (Figure 4).

## 5. Nuclear Antigens as a Therapeutic Target

To prevent the release of nuclear antigens such as histone H1 and HMGB1, resulting in the activation of innate and adaptive immune responses, several strategies have been proposed. The therapeutic potential of anti-HMGB1 antibody, soluble RAGE, and anti-RAGE neutralizing antibody has been demonstrated in experimental sepsis [73, 74], liver ischemia/reperfusion injury [75], Con-A-induced hepatic injury [76], traumatic brain injury [77], and organ transplantation [78, 79]. The therapeutic potential of antihistone H1 polyclonal antibody for overcoming rejection and liver inflammation was also confirmed by our group [19, 40]. We also confirmed the great potential of histone H1 vaccination in transplant recipients for tolerance induction [80, 81]. To further explore the roles of histone H1 and its future clinical application, we have generated antihistone H1 monoclonal antibodies (clone: 16G9, IgM), which possess immunosuppressive activity *in vitro* [82]. In addition, we have identified the functional epitope (SSVLYGGPPSAA) responsible for the immunosuppressive activity of 16G9 and have confirmed the diagnostic and therapeutic potential of histone H1 peptide [83]. In addition to neutralizing antibody therapy, an HMGB1 absorption column (polymyxin B-immobilized fibers) has been developed and clinically applied for the removal of circulating HMGB1 in patients with septic shock, acute respiratory distress syndrome, and idiopathic pulmonary fibrosis with acute exacerbation [84–90]. The therapeutic potential of HMGB1 antagonists such as HMGB1 A box peptide has also been reported [91, 92].

## 6. Summary and Future Directions

In this review, we have discussed the diagnostic and therapeutic potential of nuclear antigens (histone H1 and HMGB1) and the corresponding antinuclear Abregs on infection, injury, inflammation, and transplant rejection. One of the immunosuppressive mechanisms of antinuclear Abregs is the direct binding of circulating nuclear antigens, which triggers the immune response (Figure 1). In addition, our previous study strongly suggested the binding of antihistone H1 Abregs to histone H1-like molecules, which may be transiently expressed on the cell membrane of splenocytes [19]. We have also demonstrated that antihistone H1 Abregs may selectively suppress the MAPK, NF-*κ*B, and calcineurin-NFAT signaling pathways during T-cell activation [40], coordinate the Th1/Th2 balance [81], and induce CD4^+^CD25^+^ T cells [65]. Recent evidence suggests that antihistone H1 Abregs negatively regulate the harmful T-cell response, in part through collaboration with Tregs [93]. Although further investigation is needed, the direct effects of nuclear antigens and corresponding antinuclear Abregs on immune cells may play important roles in inflammation, rejection, and tolerance induction. Interestingly, the induction of antinuclear Abregs (i.e., the autoimmune response against nuclear antigens) may suppress alloantibody production during rejection after OLT (Figure 3). A crucial issue is why cell death-associated moieties and corresponding autoantibodies, which elicit clinical autoimmunity in patients with autoimmune diseases, could be indispensable for immune regulation in other settings. In our previous study, nuclear histone H1 and Freund's complete adjuvant were injected into naive rats and resulted in different autoantibody responses against histone H1 in tolerogenic PVG OLT recipients and rejecting LEW OLT recipients [68]. The transient induction of autoantibodies in normal mice challenged with dying cells and adjuvants (Freund's incomplete adjuvant or DCs) was also reported without clinical or histological features of autoimmunity, while clinical autoimmunity develops in autoimmune-prone mice [94, 95]. Therefore, we speculate that the response to dying cells in OLT recipients may be one of the key factors determining the clinical outcome. How to modulate the balance between autoimmunity and alloimmunity is an important issue for the extrinsic regulation of unwanted immune responses and the induction of immune tolerance (Figure 4). Our present data also reveal the diagnostic significance of nuclear antigens for the prediction of acute rejection after liver transplantation (Figure 2). The development of fast, accurate, and precise diagnostic tools by measuring the blood level of nuclear histone H1 and HMGB1 would allow clinicians to evaluate immune status and modulate the dose of immunosuppressants for rejection control. The development of absorption columns for circulating nuclear antigens (histone H1 and HMGB1) as well as neutralizing humanized monoclonal antibodies may help to establish novel immunotherapies for infection, injury, inflammation, and transplant rejection.

## Abbreviations

HMGB1: High-mobility group box 1
DA: Dark Agouti
PVG: Piebald Virol Glaxo
OLT: Orthotopic liver transplantation
DCs: Dendritic cells
LSF: Liver suppressor factor
AMR: Antibody-mediated rejection
Tregs: Regulatory T cells
Con A: Concanavalin A
DAMPs: Damage-associated molecular pattern molecules
RAGE: Receptor for advanced glycation endproduct
TLR: Toll-like receptor
NSCLC: Nonsmall cell lung cancer.
